# Awareness, knowledge, use, willingness to use and need of Pre-Exposure Prophylaxis (PrEP) during World Gay Pride 2017

**DOI:** 10.1371/journal.pone.0204738

**Published:** 2018-10-19

**Authors:** Carlos Iniesta, Débora Álvarez-del Arco, Luis Miguel García-Sousa, Belén Alejos, Asunción Díaz, Nieves Sanz, Jorge Garrido, Michael Meulbroek, Ferran Pujol, Santiago Moreno, María José Fuster-Ruiz de Apocada, Pep Coll, Antonio Antela, Jorge del Romero, Oskar Ayerdi, Melchor Riera, Juanse Hernández, Julia del Amo

**Affiliations:** 1 National Center of Epidemiology, Carlos III Health Institute, Madrid, Spain; 2 Universidad Complutense de Madrid, Madrid, Spain; 3 La Doctora Álvarez Communication Agency, Madrid, Spain; 4 Asociación CoRIS, Madrid, Spain; 5 Apoyo Positivo, Madrid, Spain; 6 Projecte dels NOMS, Hispanosida, Barcelona, Spain; 7 Hospital Universitario Ramón y Cajal, Madrid, Spain; 8 Universidad Alcalá de Henares, Madrid, Spain; 9 Seisida, Madrid, Spain; 10 AIDS Research Institute-IrsiCaixa, Barcelona, Spain; 11 Hospital Clinico Universitario de Santiago de Compostela, Infectious Diseases Department, Santiago de Compostela, Spain; 12 Centro Sanitario Sandoval IdISSC, Unidad ITS/VIH, Madrid, Spain; 13 Hospital Son Espases, Palma de Mallorca, Spain; 14 Grupo de Trabajo sobre Tratamientos del VIH -gTt-VIH, Barcelona, Spain; British Columbia Centre for Excellence in HIV/AIDS, CANADA

## Abstract

**Objective:**

To assess the awareness, knowledge, use, and willingness to use and need of PrEP among men who have sex with men (MSM) and transgender women (TW) who attended World Gay Pride (WGP) 2017 in Madrid.

**Design and methods:**

Online survey. Participants were recruited through gay-oriented dating apps and HIV Non-Governmental Organizations´ social media. Inclusion criteria included being MSM or TW, age 18 years old or above, and having attended WGP in Madrid. Information regarding the participant’s awareness and knowledge, use or willingness to use, and need for PrEP was collected, as well as sociodemographic characteristics. Participants were considered to be in need of PrEP if they met one of the following indication criteria: having practiced unprotected anal intercourse with more than 2 partners, having practiced chemsex, or having engaged in commercial sex—all in the preceding 6 months. Descriptive and multivariable analyses with logistic regression were conducted.

**Results:**

472 participants met the inclusion criteria and completed the questionnaire. The mean age was 38, 97.7% were MSM, 77% had a university education, and 85% were living in Spain, mostly in big cities. Overall, 64% of participants were aware of PrEP, but only 33% knew correctly what PrEP was. 67% of HIV-negative participants were willing to take PrEP, although only 5% were taking it during WGP, mostly due to lack of access. 43% of HIV-negative respondents met at least one PrEP indication criteria. For HIV-negative men living in Spain, university education and living in big cities was associated with PrEP awareness. Lower education level and meeting PrEP criteria was associated with willingness to use PrEP.

**Conclusions:**

Our study shows that among MSM attending WGP 2017 in Madrid, there was limited PrEP awareness, low accuracy of PrEP knowledge, and a high need and willingness to use PrEP. Health authorities should strengthen existing preventive strategies and implement PrEP.

## Introduction

Sexually transmitted HIV remains an important public health issue in Spain. In 2016, out of the 3353 new HIV diagnoses, 53.1% were men who have sex with men (MSM). While HIV incidence has steadily decreased around the globe since 2009, it has remained stable for MSM at rates ranging from 10–12 new cases per 100,000 men [1].

In order to reverse this trend, the Spanish National AIDS Plan recommends promoting condom use, HIV testing, rapid access to Antiretroviral Therapy (ART), and Post-exposure Prophylaxis (PEP) [2]. However, it has been strongly demonstrated that condoms are not used consistently among some groups of MSM who are at high risk of being infected with HIV [3,4]. Overall, undiagnosed HIV is estimated to be between 14% and 22% of people living with HIV [5]. Although PEP is available and free in public hospital’s emergency rooms, there is a lack of awareness and use of it [6].

In this context, Pre-Exposure Prophylaxis (PrEP) could be an additional and effective preventive measure that, unfortunately, is not yet formally available in Spain. The pre-exposure prophylactic use of the commercial combination of tenofovir/emtricitabine has been approved by the European Medicines Agency [7] and has already been implemented in European countries with epidemiological patterns similar to Spain’s [8]—showing to be effective, safe, and cost-effective [9–12]. In Spain, the Agency of Medicines and Medical Devices (AEMPS) has also approved the commercial combination of tenofovir/emtricitabine for PrEP indication as part of a comprehensive prevention program [13]. The National Ethics Committee issued a statement in favour of PrEP public funding in March 2017 [14]. In June 2016, the Spanish Scientific Clinical Society for AIDS (GeSIDA) issued a PrEP recommendation document to facilitate the way forward [15], followed by another from the National AIDS Plan at the Ministry of Health (MoH) [16]. These documents recommend PrEP for populations most at risk of acquiring HIV, especially MSM and transgender women (TW). However, the Spanish MoH and its equivalent health authorities in the Autonomous Communities have not implemented PrEP, thus it is not available within the Spanish Health System [17].

Additionally, Madrid was hosting the celebration of World Gay Pride (WGP) between the 22nd of June and the 2nd of July 2017. Estimated attendance was 3 million people, mostly MSM, coming from a number of countries, possibly some of them prescribing PrEP. Apart from the cultural events and the official parade, many gay-oriented clubs and saunas were programing sex parties during Gay Pride week. An increased need for PrEP and an increase in PrEP use were expected among WGP attendees.

The aim of this work was to assess awareness, knowledge, use of PrEP, willingness to use PrEP, and PrEP need among MSM and TW attending WGP 2017 in the city of Madrid between June 23^nd^ and July 2^nd^.

## Materials and methods

This study was approved by the Carlos III Health Institute Research Ethics Committee.

### Design of the questionnaire

An electronic survey with a structured questionnaire was designed using questionnaires from previous PrEP studies [11], the Spanish Sexual Health Survey [18], and GeSIDA PrEP Guidelines [15]. It was reviewed by a group of 9 experts. The questionnaire included a welcome message in which participants were informed about the objectives of the study and were asked for their consent to participate. The survey was advertised through gay-oriented dating apps and the social media of HIV Non-Governmental Organizations (NGO) June 26^th^ and August 8^th^ of 2017, showing the following messages: “Are you PrEPared for the pill that prevents HIV? / Help us to improve the health of LGBT community by answering some questions / Are you PrEPared for World Pride?” The questionnaire was available in Spanish, English, Russian, Italian, and French. Participants self-completed the questionnaire. Inclusion criteria for participants was: being MSM or TW, over 18 years old, and having attended WGP in Madrid.

### Definition of variables

Information collected in the questionnaire permitted the creation the five variables of interest (PrEP awareness, PrEP knowledge, PrEP use, willingness to use PrEP, and PrEP need) and the variables of exposure: age, gender identity, sexual orientation, educational level, place of residence, HIV status, and number of inhabitants in place of residence (only for those living in Spain). Participants were considered MSM if they were cisgender men who had sex with cisgender men.

PrEP awareness was assessed by asking participants if they knew of “the so-called Pre Exposure Prophylaxis (PrEP)”. PrEP knowledge was assessed using multiple true-false questions in which participants had to mark true among the following: i) It is a pill; ii) It reduces the risk of getting HIV. The other true-false questions were: iii) It is an injection; iv) It can be used after you believe you have had high-risk sex with the possibility of becoming infected with HIV; v) It reduces the risk of getting HIV and other sexually transmitted infections (STI). Only participants who marked options i and ii as true and did not mark options iii, iv, and v as true were considered to have accurate knowledge of PrEP.

PrEP was described as “a pill that, if you take it, protects you from getting HIV even if you have sex with a person who has HIV. PrEP does not protect against other STI, only HIV” to respondents who were not aware of PrEP and to respondents who were aware of PrEP but had never used it.

We considered participants to be in PrEP need if they met one of the following indication criteria: having practiced unprotected anal intercourse with more than 2 partners, having practiced Chemsex, or having engaged in commercial sex—all in the preceding 6-months as recorded in GeSIDA PrEP Guidelines [15]. Other PrEP indication criteria, such as STI diagnosis or PEP use, were not considered due to the difficulties of self-reporting these variables and to ensure completion of the questionnaire by keeping it brief. Data are available in the supporting information S1 File.

### Sampling, size of the simple and analysis strategy

We estimated that a sample size of 385 participants would be needed in order to assess PrEP use considering an expected PrEP use of 50% (worst-case scenario) and a bilateral precision of five points.

Descriptive analyses were conducted for all interest variables (PrEP awareness, PrEP knowledge, PrEP use, willingness to use PrEP, PrEP need) and exposure variables. Chi-square, Fisher’s exact tests, and Wilcoxon rank-sum tests were used to identify differences between four of the main outcomes: PrEP awareness, PrEP use and willingness to use PrEP (collapsed in one single variable as it was considered that being in use of PrEP was equivalent to be willing to use PrEP) and PrEP need. Multi-variable logistic regression was developed in order to identify which variables of exposure were associated with PrEP awareness, PrEP use or willingness to use, and PrEP need for a subsample of MSM living in Spain. PrEP awareness was considered as a variable of exposure for PrEP use or willingness to use and for PrEP need. Likewise, PrEP need was considered a variable of exposure for PrEP use or willingness to use. HIV+ individuals were not considered in the analysis of factors associated with PrEP need and use or willingness to use PrEP.

We used multivariable logistic regression to calculate odds ratios (OR) and 95% confidence intervals (CI) for the association between the three main outcomes and predictive factors. An explanatory modelling was performed following a stepwise backward strategy, following the Akaike Information Criterion (AIC) and the Wald Test p-values. We selected the model with highest significance (Wald test p-values under 0.05) and the best goodness-of-fit (lowest AIC value) [19]. The assumptions for the logistic regression model were checked. All statistical analyses were performed using Stata software (version 14.0; Stata Corporation, College Station, Texas, USA).

## Results

Of the 1,269 people that accessed the questionnaire, 1,006 completed it and 472 met the inclusion criteria. The most frequent exclusion criteria was not attending WGP (379), followed by not consenting to participate in the study after being asked (114). Participants who did not complete the questionnaire were significantly older than those who did. No other information was available for this group (data not shown). Almost all respondents were MSM, most of whom were living in Spain and had a university education. The majority of participants declared they did not live with HIV (Table 1).

**Table 1:** Characteristics of the sample (n = 472).

	% (n)
**Age (m, SD)**	38 (9.44)
**Age groups**	
18–25 years old	9.3 (44)
26–25 years old	31.6 (149)
36–45 years old	35.0 (165)
More than 45 years old	24.2 (114)
**Gender identity**	** **
Cisgender Man	98.5 (465)
Transgender Man	0.6 (3)
Transgender Woman	0.4 (2)
Other	0.4 (2)
MSM*	** **
Yes	97.7 (461)
No	2.3 (11)
**Educational level**	** **
University or more	77.3 (107)
High school or less	22.7 (365)
**Place of residence**	** **
Spain	85.4 (403)
Latin America or Caribbean	3.0 (14)
Another countries in Europe	10.2 (48)
North America	0.4 (2)
Unknown	1.1 (5)
Inhabitants in place of residence**	** **
More than 500,000	66 (266)
Between 100,000 and 500,000	12.7 (51)
Between 50,000 and 100,000	7.0 (28)
Between 10,000 and 50,000	6.0 (24)
Under 10,000	7.4 (30)
Unknown	1.0 (4)
**HIV status**	** **
PLHIV	14.2 (67)
Not living with HIV	71.8 (339)
Don't know	11.4 (54)
Prefer not to say	2.5 (12)
**Are aware of PrEP**	
Yes	64.19 (303)
No	35.81 (169)
**Know what PrEP is** ***	
Yes	32.63 (154)
No	31.57 (149)
Not aware of PrEP	35.81 (169)
**Need PrEP** ****	** **
Yes	42.5 (144)
No	57.5 (195)

* Considering MSM as cisgender men that have sex with another cisgender men.

**Among those living in Spain.

***Among those being aware of PrEP

**** Meet PrEP criteria (among those who reported not be living with HIV)

### PrEP awareness and knowledge

About two out of 3 participants (64%) were aware of PrEP, but only 33% correctly knew what PrEP was. Higher educational levels and living in cities with larger populations were independently associated with PrEP awareness among HIV-negative men living in Spain (Table 2).

**Table 2:** Variables associated to PrEP awareness, use or willingness to use PrEP, and need of PrEP for MSM living in Spain.

	Are aware of PrEP (n = 397)			Are using or willing to use PrEP (n = 286) *			PrEP need (n = 286) *		
	% (n)	p value	aOR (IC 95%)	% (n)	p value	aOR (IC 95%)	% (n)	p value	aOR (IC 95%)
**Age**	64.1 (25)	0.379	…			** **			
18–25 years old	69.05 (87)	0.379	…	48 (12)	0.075	…	48 (12)	0.836	…
26–35 years old	60.81 (90)		…	64.6 (62)		…	39.2 (38)		…
36–45 years old	58.33 (49)		…	69.7 (76)		…	38.5 (42)		…
More than 45 years old				76.4 (42)		…	41.8 (23)		…
**Education**	46.15 (42)	<0.001	Reference	** **	** **	** **		** **	
High school or less	68.3 (209)	<0.001	2.64 (1.50–4.67)	78.3 (54)	0.028	Reference	47.1 (33)	0.207	…
University or more				63.9 (138)		0.52 (0.27–0.99)	38.0 (82)		…
**Inhabitants in place of residence**	69.35 (181)	<0.001	Reference	** **	** **	** **		** **	…
More than 500,000	44 (22)	<0.001	0.35 (0.17–0.71)	64.7 (119)	0.230	…	39.7 (73)	0.884	…
						
Between 500,000 and 100,000	53.66 (44)		0.44 (0.24–0.82)	78.1 (32)		…	39.0 (16)		…
Less than 100,000				70.2 (40)		…	43.1 (25)		…
**HIV status**	61.54 (176)	0.002	…						
Not living with HIV	83.02 (44)	0.002	…	…	…	…	…	…	…
PLWHIV	47.92 (23)		…	…	…	…	…	…	…
Don't know	80 (8)		…	…	…	…	…	…	…
Prefer not to say				…	…	…	…	…	…
**Are aware of PrEP**	…	…	…						
Yes	…	…	…	64.6 (113)	0.204	…	41.5 (73)	0.580	…
No				71.8 (79)		…	38.2 (42)		…
**Need PrEP**	…	…	…	** **	** **	** **	** **	** **	** **
No	…	…	…	79.8 (91)	<0.001	Reference	…	…	…
Yes	64.1 (25)	0.379	…	59.1 (101)		2.67 (1.54–4.65)	…	…	…

* Participants who reported to be living with HIV were not considered

### PrEP use and willingness to use

Only HIV-negative participants aware of PrEP (n = 215) were asked about PrEP use. Among them, 17 (8%) used PrEP during WGP, mainly prescribed by a doctor (n = 6) or purchased on the internet (n = 4). Other modes of acquisition (n = 7) included enrolment in clinical trials, emergency rooms, or through a prescription for indications other than PrEP, largely PEP. Only 9 out of the 17 PrEP users were living in Spain. The remaining 198 participants did not use PrEP during WGP due to a lack of access (45%), a lack of willingness (39%), and other reasons (16%).

In total, 67% of HIV-negative participants were using or would use PrEP. For men living in Spain, use of or willingness to use PrEP was 68%. Willingness to use PrEP was high among HIV-negative participants: 63% of them (n = 212) would use PrEP, mainly to prevent HIV (53%), to have unprotected sexual intercourse (14%), or to feel safer during sex (8%). The main reasons to reject PrEP (n = 112) were condom preference (22%), lack of prevention of other STIs (19%), or having a steady partner (13%). Only two participants reported not wanting to use PrEP for economic reasons. In the multivariable analyses, meeting PrEP criteria (aOR = 2.67) and not having a university education (aOR = 0.52) were independently associated to using or willingness to use PrEP (Table 2).

### PrEP need

Of the 339 participants not living with HIV, 43% met at least one of the PrEP indication criteria considered in this study [12]. Among them, 84% had unprotected anal intercourse with three or more partners, 51% practiced chemsex, and 16% engaged in commercial sex in the previous 6 months (these categories are not mutually exclusive). We did not find any statistically significant difference in PrEP indication criteria regarding age, education, number of inhabitants in place of residence, and PrEP awareness (Table 2).

## Discussion

Awareness was higher among people living with HIV (PLHIV), people with university education, and people living in big cities. This may reflect barriers to access information among MSM with lower education levels and MSM who live in smaller cities. This creates a situation of increased vulnerability, which has already been described for theLGBT community. For example, there is a relationship between low education levels and late HIV diagnosis across different European countries [20]. Additionally, we did not reach a sufficient number of TW to extract conclusions about this group. This reflects higher difficulty in reaching this population, and the need to conduct specific studies to assess how PrEP implementation strategies would work for TW. All these determinants, which possibly foster health inequalities, need to be taken into account when developing PrEP implementation policies.

Regarding overall PrEP awareness, recent studies in European contexts similar to ours show different values: 42% for MSM attendees at Gay Pride in Lisbon 2010 [21]; 85% for MSM users of sex-dating applications in London [22]; 54% for HIV- MSM from the Amsterdam Cohort Study for AIDS and HIV in 2015 [23]; 35% for MSM who had unprotected anal intercourse in 2016 from Scotland, Wales, and Ireland [24]. Although these data refer to prior PrEP implementation contexts, differences across these European cities may be explained by local factors, such as support for PrEP from community and administration levels. In Spain, our results were higher than those reported by the Ferrer et al. study, who found a PrEP awareness of 29% for MSM recruited online or in HIV testing clinics in Spain 2013–2014 [25]. Despite the increase in PrEP awareness, PrEP knowledge was low. From our point of view, this could be related to the fact that very few official information campaigns have been conducted by the Ministry of Health or other health authorities within the last few years—neither informing the public about PrEP strategy itself or its political and legal situation, which still remains unclear.

However, we found a considerably high willingness to use PrEP and (like awareness) it was higher than the one assessed by Ferrer et al (58%) in Spain 2013–14 [26]. This number is problematic since PrEP knowledge was poor in our sample and the information provided was limited to participants who were not aware of PrEP. This limitation could have overestimated the willingness to use PrEP: we did not know if participants would have responded differently had they been better informed about possible side effects or had greater knowledge of PrEP. Despite this limitation, we observed that people who met PrEP criteria were more likely to be willing to use PrEP. This demonstrates that, despite low knowledge, the most at-risk people could identify PrEP as a useful strategy to reduce the risk of being infected with HIV. This information could facilitate a strategy to reach the most at-risk groups when PrEP is approved in Spain. The relationship between PrEP indication criteria and risk of acquiring HIV has already been shown by Ayerdi et al, who found that a high proportion of new HIV cases diagnosed in 2014 at the most important STI clinic in Madrid met PrEP criteria [27]. The relationship between high-risk behavior and willingness to use PrEP has also been shown before [22,23,27].

Although most of the participants were willing to use PrEP, 33% of them were not. Having a university education was associated to lower use of or willingness to use PrEP. Further research is needed to understand this association that has been described in similar contexts [28], as well as for other factors that could lead to less willingness to use PrEP. Our findings suggest that PrEP may be perceived as less useful for MSM of higher socio-economic status. It is necessary to understand if PrEP-related stigma, described in similar contexts [29] could be related to this perception.

Since participants were self-selected, people who participated in this survey could have greater knowledge or willingness to use PrEP (selection bias). Awareness, knowledge, use of, and willingness to use PrEP could be overestimated in our study. We acknowledge that we asked about unprotected sex instead of condomless sex, which could overestimate the proportion of people having condomless sex as participants replied using their own definition of unprotected sex. Furthermore, we did not take not into account other GeSIDA indication criteria (diagnosis of STI, administration of PEP, serodiscordant couple without clinical monitoring, and injected drug use within last 6 months) [15] which could underestimate the number of people at high-risk of being infected with HIV during WGP. Nevertheless, to our knowledge this is the first work in Spain that correlates a willingness to use PrEP with meeting PrEP criteria, and the first to address PrEP need among attendees to WGP celebration. This work could help implement a PrEP strategy and could ease the planning of upcoming, similar events.

In summary, the number of participants who met PrEP criteria was high, indicating that the risk of acquiring HIV is equally and worryingly high. Although knowledge of PrEP was limited, a high proportion of MSM living in Spain, especially those most at-risk of acquiring HIV, was willing to use PrEP. Health authorities should strengthen existing preventive strategies and implement PrEP in order to reduce the number of new HIV cases.

## Supporting information

S1 File Data set. (DTA)
