# Correction: Awareness, knowledge, use, willingness to use and need of Pre-Exposure Prophylaxis (PrEP) during World Gay Pride 2017

**DOI:** 10.1371/journal.pone.0216376

**Published:** 2019-04-29

**Authors:** Carlos Iniesta, Débora Álvarez-del Arco, Luis Miguel García-Sousa, Belén Alejos, Asunción Díaz, Nieves Sanz, Jorge Garrido, Michael Meulbroek, Ferran Pujol, Santiago Moreno, Maria Jose Fuster-RuizdeApodaca, Pep Coll, Antonio Antela, Jorge del Romero, Oskar Ayerdi, Melchor Riera, Juanse Hernández, Julia del Amo

The 11^th^ author’s name is spelled incorrectly. The correct name is: Maria Jose Fuster-RuizdeApodaca.

In the Design of the questionnaire subsection of the Materials and methods, there is an error in the fourth sentence. The correct sentence is: The survey was advertised through gay-oriented dating apps and the social media of HIV Non-Governmental Organizations (NGO) between June 26^th^ and August 8^th^ of 2017, showing the following messages: “Are you PrEPared for the pill that prevents HIV? / Help us to improve the health of LGBT community by answering some questions / Are you PrEPared for World Pride?”

In Table 2, the rows are misaligned. Please see the complete, correct Table 2 here.

**Table 2:** Variables associated to PrEP awareness, use or willingness to use PrEP, and need of PrEP for MSM living in Spain.

	Are aware of PrEP (n = 397)			Are using or willing to use PrEP (n = 286) *			PrEP need (n = 286) *		
	% (n)	p value	aOR (IC 95%)	% (n)	p value	aOR (IC 95%)	% (n)	p value	aOR (IC 95%)
**Age**						** **			
18–25 years old	64.1 (25)	0.379	…	48 (12)	0.075	…	48 (12)	0.836	…
26–35 years old	69.05 (87)		…	64.6 (62)		…	39.2 (38)		…
36–45 years old	60.81 (90)		…	69.7 (76)		…	38.5 (42)		…
More than 45 years old	58.33 (49)		…	76.4 (42)		…	41.8 (23)		…
**Education**				** **	** **	** **		** **	
High school or less	46.15 (42)	<0.001	Reference	78.3 (54)	0.028	Reference	47.1 (33)	0.207	…
University or more	68.3 (209)		2.64 (1.50–4.67)	63.9 (138)		0.52 (0.27–0.99)	38.0 (82)		…
**Inhabitants in place of residence**				** **	** **	** **		** **	…
More than 500,000	69.35 (181)	<0.001	Reference	64.7 (119)	0.230	…	39.7 (73)	0.884	…
Between 500,000 and 100,000	44 (22)		0.35 (0.17–0.71)	78.1 (32)		…	39.0 (16)		…
Less than 100,000	53.66 (44)		0.44 (0.24–0.82)	70.2 (40)		…	43.1 (25)		…
**HIV status**									
Not living with HIV	61.54 (176)	0.002	…	…	…	…	…	…	…
PLWHIV	83.02 (44)		…	…		…	…		…
Don't know	47.92 (23)		…	…		…	…		…
Prefer not to say	80 (8)		…	…		…	…		…
**Are aware of PrEP**									
Yes	…	…	…	64.6 (113)	0.204	…	41.5 (73)	0.580	…
No	…		…	71.8 (79)		…	38.2 (42)		…
**Need PrEP**				** **	** **	** **	** **	** **	** **
No	…	…	…	79.8 (91)	<0.001	Reference	…	…	…
Yes	…		…	59.1 (101)		2.67 (1.54–4.65)	…		…

* Participants who reported to be living with HIV were not considered

Reference 2 is spelled incorrectly. The correct reference is: Ministerio de Sanidad, Servicios Sociales e Igualdad. Plan Estratégico de Prevención y Control de la infección por el VIH y otras infecciones de transmisión sexual [Internet]. 2013. Available: https://www.msssi.gob.es/ciudadanos/enfLesiones/enfTransmisibles/sida/docs/PlanEstrategico2013_2016.pdf

The URL in reference 3 is incorrect. The correct URL is: https://ecdc.europa.eu/sites/portal/files/media/en/publications/Publications/EMIS-2010-european-men-who-have-sex-with-men-survey.pdf

The URL in reference 13 is incorrect. The correct URL is: https://cima.aemps.es/cima/pdfs/ft/04305001/FT_04305001.pdf

The URL in reference 17 is incorrect. The correct URL is: http://www.mscbs.gob.es/ciudadanos/enfLesiones/enfTransmisibles/sida/docs/PROFILAXIS_PREEXPOSICION_VIH.pdf

Ferrer L, Folch C, Fernandez-Davila P, Garcia A, Morales A, Belda J, et al. was incorrectly included as reference 26. As a result, all subsequent references are misnumbered. References 27–29 should be references 26–28.
